# Measurement of intraarticular tibiofemoral rotation in routine MRI is not reliable: A comparison of knee joints in torsional and routine MRI

**DOI:** 10.1002/jeo2.70845

**Published:** 2026-07-07

**Authors:** Sina Gräber, Annett Klinder, Johanna Lippuner, Felix Hüttner, Andrzej Jasina, Parisa Pourostad, Elizabeth Arendt, Thomas Tischer, Jörg Harrer, Felix Ferner, Christoph Lutter

**Affiliations:** ^1^ Department of Orthopaedic Surgery Rostock University Medical Center Rostock Germany; ^2^ Department of Orthopaedic Surgery Sanaklinikum Lichtenfels Lichtenfels Germany; ^3^ Department of Orthopaedic Surgery University of Minnesota Minneapolis USA; ^4^ Department of Orthopaedic Surgery Malteser Waldkrankenhaus St Marien Erlangen Germany

**Keywords:** intraarticular tibiofemoral rotation, knee version, torsional MRI, knee MRI, measurement reliability

## Abstract

**Purpose:**

Intraarticular tibiofemoral (iaTF) rotation at the axial plane of the knee is considered as one parameter of joint alignment. Its measurement is originally described on fully extended legs in torsional computed tomography/magnetic resonance imaging (CT/MRI). The aim of this study was to investigate whether the measurement of iaTF rotation is reliably possible in routine MRIs.

**Methods:**

Patients who underwent torsional MRI (torMRI) and routine MRI (rouMRI) of the knee were retrospectively analysed. iaTF rotation corresponds to the angle between a tangent to the dorsal femoral condyles and a tangent to the dorsal tibial plateau in the axial plane (positive values indicating external‐/negative values internal rotation). In rouMRI, the flexion angle between the bony axes in the sagittal plane was measured. Spearman correlation coefficient (r_S_) and intraclass correlation coefficient (ICC) were calculated assessing the agreement of iaTF rotation between imaging modalities. Cases showing a deviation of 3° or less were summarised as “group‐with‐good‐agreement”. Sensitivity and specificity for assignment to the group‐with‐good‐agreement based on the flexion were calculated using ROC‐analysis.

**Results:**

90 cases (78 patients, age 25.6 ± 9.6 years) were included. iaTF rotation was 5.7° ± 6.7° in torMRI and 1.0° ± 8.0° in rouMRI. The measurements differed by an absolute value of 6.4° ± 4.6°. Strong positive correlation (*r*_s_ = 0.591; *p* < 0.001) and moderate agreement (ICC = 0.688) were calculated between iaTF rotation measurements. Flexion in rouMRI (7.1 ± 6.1°) correlated moderately positively with the difference in iaTF rotation between torMRI and rouMRI. The group‐with‐good‐agreement showed a significantly lower flexion (*p* = 0.022). Assuming that lower flexion in rouMRI predicts good agreement, the ROC analysis yielded a threshold of 4.65° flexion (model quality = 0.53). For cases below the threshold, sensitivity was 74.2% and specificity 54.5% for correct assignment to the group‐with‐good‐agreement.

**Conclusion:**

Intraarticular TF rotation is not reliable measurable on routine MRIs with no uniform protocol, as the results deviate considerably from those in the torsional MRIs.

**Level of Evidence:**

Level III.

**Abbreviations**
CT: computed tomography
DFC: dorsal femoral condyle tangent line
DTH: dorsal tibial head tangent line
iaTF rotation: intraarticular tibiofemoral rotation
ICC: intraclass correlation coefficient
max: maximum
min: minimum
mm: millimetre
MRI: magnetic resonance tomography
n: number
ROC: receiver operating characteristic
rouMRI: routine magnetic resonance tomography
r_S_
: Spearman correlation coefficient
SD: standard deviation
torMRI: torsional magnetic resonance tomography
TT‐TG: tibial tubercle to trochlea groove
y: years

## INTRODUCTION

Intraarticular tibiofemoral (iaTF) rotation of the knee joint, that is, rotation between the distal femur and the proximal tibia in the axial plane, is increasingly the subject of research. Recent studies demonstrated that an increased iaTF rotation could lead to increased malalignment of the knee joint and is associated with patellofemoral instability, torsional deformity of the lower limb and graft failure after anterior cruciate ligament reconstruction [3, 8, 13, 18, 22, 23, 33, 35, 36]. Eckhoff et al. established the term “knee version” which is equivalent to iaTF rotation, based on a computed tomography (CT) study describing a mean value of 1° external rotation of the knee joint as physiological [10]. Huettner et al. confirmed the findings by Eckhoff et al. by determining an average knee version of 1.3° ± 3.9° external rotation in an magnetic resonance imaging (MRI) study on healthy individuals [10, 16]. Both, torsional CT and MRI analysis provide reproducible and comparable values for torsional analysis [19, 26, 29, 30]. As the iaTF rotation is normally measured in torsional CT or MRI, the normal values refer to a fully extended knee joint [10, 16]. Theoretically, it is possible to measure iaTF rotation in every routine MRIs, as long as the femoral condyles and the dorsal tibial plateau are depicted. However, in most routine MRI examinations, the knee joints are slightly flexed due to the use of MRI coils. This is likely to cause a change in the iaTF rotation due to the screw‐home mechanism—that is, tibial external rotation during end extension—of the knee joint [28]. The data on the extent of the screw‐home mechanism vary in the literature between 7° and 20° tibial external rotation during extension of the knee joint from up to 160° flexion to 0° [15, 21, 28].

The present study aims to determine whether iaTF rotation can be reliably measured on routine knee MRI when compared with torsional MRI among patients with existing patellar instability and torsional deformity. Given that routine MRI is typically performed with a slightly flexed knee, we hypothesise that this positioning does not generally allow for reliable assessment of iaTF rotation.

However, we secondly hypothesise that when knee flexion during routine MRI is minimal and approximates the position used in torsional MRI, the resulting measurements may achieve comparable reliability. Therefore, this study seeks to identify a threshold of knee flexion below which routine MRI provides sufficiently reliable measurements of iaTF rotation.

## METHODS

### Patient selection and MRI assessment

In this single‐center study, patients who received a torsional MRI between 2019 and 2024 due to existing patellar instability or patellofemoral maltracking with an existing torsional deformity of the lower limb prior to surgical treatment were included. Further inclusion criteria were a routine MRI of the knee joint and MRI‐morphologically closed physis. An exclusion criterion was any type of surgery performed on the affected knee between the MRI scans. Ethical approval was obtained by the local ethics committee.

Torsional MRI analysis were all performed according to the same previously published protocol with a 1.5‐T MRI scanner (Magnetom Area; Siemens) with automatic table positioning. During the 12‐min evaluation time, 30 slices with 5 mm thickness in the axial plane were obtained from hip, knee and ankle joints each on both sides. Patient positioning was supine and feet forward with hip, knee and ankle fixed in neutral position using a uniform positioning device [16]. Routine MRIs were acquired from several radiology departments and did not undergo an uniform protocol. Measurements on torsional and routine MRI were each carried out by two trained orthopaedic surgeons using a commercial picture archiving and communication system (DeepUnity Diagnost Version 2.0.2.2, Daedalus DACH). In the torsional MRI, femoral torsion (angle between the proximal femoral axis and the dorsal femoral condyle tangent line [DFC]) and tibial torsion (angle between the dorsal tibial head tangent line [DTH] and the distal tibial axis) were measured according to Waidelich et al. [34]. In accordance with this method, DFC was drawn along the maximum dorsal points of the femoral condyles, immediately proximal to the knee joint plane. The DTH was placed just distal to the knee joint plane, along the maximum proximal extension of the tibial plateau [34]. IaTF rotation refers to the angle between DFC and DTH [10, 16].

**Figure 1:**
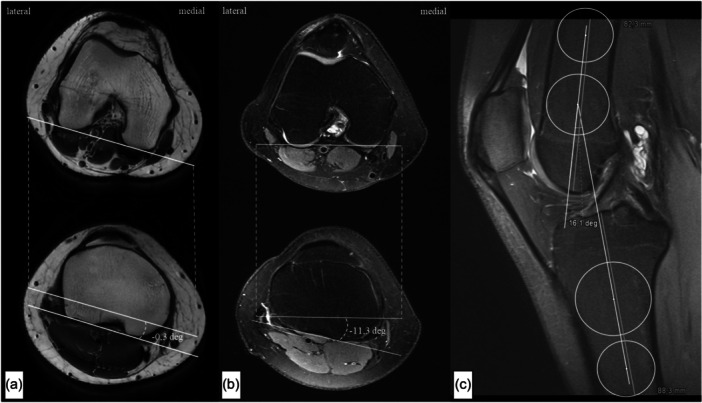
(a) iaTF rotation measurement in torsional MRI: Angle between DFC and DTH. (b) iaTF rotation measurement in routine MRI: Angle between DFC and DTH (c) Determination of femoral and tibial length in routine knee MRI and flexion measurement (the bony axes are defined by the centres of the circles). DFC, dorsal femoral tangent line (defined by the most posterior points of the femoral condyles); DTH, dorsal tibial tangent line (defined by the most posterior point of the proximal tibial plateau); iaTF, intraarticular tibiofemoral; MRI, magnetic resonance imaging [16, 34].

Analogous to the torsional MRI, iaTF rotation in routine MRI was determined as the angle between DFC and DTH. The flexion angle between the femur and tibia in the sagittal plane was measured in routine MRI in the layer in which the anterior cruciate ligament presented best. For this purpose, two circles each were drawn in the tibia and the femur, with the circle edges adjacent to the outer cortical bone. A line was drawn through the centres of the circles, which was defined as the respective femur or tibia axis. The flexion angle was determined between these two axes. This method was modified according to Griffith et al. [14]. In addition, the length of the femur and tibia were measured in each routine MRI scan. The entire measurements which were performed in the routine MRI are illustrated in Figure 1.

### Calculation of the mean difference in iaTF rotation between the two imaging modalities

To calculate the deviation of the iaTF rotation measurements between torsional MRI and routine MRI, the iaTF rotation measured in routine MRI was subtracted from the value measured in torsional MRI for each individual case. In cases where the iaTF rotation in routine MRI was higher than in torsional MRI, this resulted in negative values. However, as the aim was to assess the degree of deviation from the reference torsional MRI only absolute values were used to classify cases with acceptable concurrence compared to cases with a deviation of more than 3° between torsional and routine MRI. The mean values of the difference in iaTF rotation between torsional MRI and routine MRI were calculated from the absolute values as well. A calculation of the mean values using positive and negative values after subtraction instead of absolute values would annihilate differences and result in an average value that is too low and therefore incorrect.

### Statistical evaluation

Statistical analysis and graphical visualisation were performed using IBM® SPSS® Statistics (v27.0.1; IBM Deutschland GmbH, Ehningen, Germany) and Microsoft® Excel (v16.78.3; Microsoft, Redmond, Washington, USA). Data were tested for normal distribution using Shapiro‐Wilk test. As the majority of data was not normally distributed non‐parametric tests (Spearman correlation analysis and Mann–Whitney *U* test) were used in the subsequent analyses if not described otherwise in the results section. Only data for iaTF rotation in rouMRI (°) were normally distributed and a t‐test was conducted to compare these measurements between cases with good and cases with insufficient agreement of iaTF rotation in torsional and routine MRI. Data are presented as median with range [minimum; maximum]. As some of the data were normally distributed we additionally reported the mathematical mean and the standard deviation in brackets (mean ± SD) to allow comparability. For correlation analysis the intraclass correlation coefficient (ICC [95% confidence interval]) for absolute agreement and the Spearman correlation coefficient (r_s_) were calculated. The interpretation of the ICC was performed according to Koo and Li [20] and that of the Spearman correlation coefficient according to Evans [11] (Table 1). A Bland–Altman plot [4, 25] was created to illustrate the consistency of the measured values between torsional and routine MRI. A deviation of 3° or less between the measurements of the iaTF rotation in torsional and routine MRI was considered an acceptable concurrence and the cases were divided into the following two groups: 'measured difference ≤ 3°'‐ group and 'measured difference > 3°'‐ group. The following measurements from the routine MRI were compared between these two groups: flexion angle and imaged length of tibia and femur, all in the sagittal plane. A ROC analysis was carried out. Based on the flexion angle measured in the sagittal plane in routine MRI, sensitivity and specificity were calculated, with which an assignment to the 'measured difference ≤ 3°'‐ group was possible. For the calculation of the ROC‐analysis and for the correlation analysis including flexion angles, all values that were in the negative range, that is, corresponding to a hyperextended knee, were excluded as it was assumed that a hyperextension is not possible during MRI scan and these values are therefore implausible.

**Table 1:** Interpretation algorithm for the correlation coefficients used in this study.

Interpretation of the correlation coefficients	
**Intraclass correlation coefficient (ICC)** [20]	
<0.50	Poor reliability
0.50–0.75	Moderate reliability
0.75–0.90	Good reliability
>0.90	Excellent reliability
**Spearman correlation coefficient (*r***_**s**_ **)** [11]	
<0.20	Very weak
0.20–0.39	Weak
0.40–0.59	Moderate
0.60–0.79	Strong
>0.80	Very strong

*Note*: The intraclass correlation coefficient was interpreted according to Koo and Li [20], and the Spearman correlation coefficient according to Evans [11].

## RESULTS

A total of 78 patients met the inclusion criteria, 12 of them had routine and torsional MRIs of both knee joints. Thus, a total of 90 knees were included. The ICC between raters was excellent for iaTF rotation in torsional and routine MRI, tibial torsion and flexion, good for femoral torsion and tibial length and moderate for femoral length (Table 2). Patients had a mean age of 25.6 ± 9.6 (12–53) years and were predominantly female (78%). The right knee (*n* = 52) was analysed more often than the left one (*n* = 38). The iaTF rotation measured in torsional MRI showed a median of 5.4° [−7.4°; 36.5°], whereas it was 1.2° [−17.6°; 21.8°] on routine MRI. The length of the recorded femur in the routine MRI was 89.2 [58.1; 386.1] mm while images contained a tibial length of 71.2 [18.2; 115.6] mm. The analysis of flexion in the sagittal plane in routine MRI revealed 7.0° [−7.9°; 23.0°]. Tables 3 and 4 provide detailed information.

**Table 2:** Interobserver reliability for measurements on torsional MRI and routine MRI.

Interobserver reliability (ICC [95% CI])	
Torsional MRI	
iaTF rotation	0.990 [0.981–0.995]
Femoral torsion	0.880 [0.806–0.926]
Tibial torsion	0.981 [0.969–0988]
Routine MRI	
iaTF rotation	0.994 [0.985–0.998]
Flexion	0.982 [0.954–0.993]
Femoral length	0.746 [0.364–0.901]
Tibial length	0.826 [0.560–0.932]

*Note*: The ICC for absolute agreement is reported, along with its 95% CI.

Abbreviations: CI, confidence interval; iaTF, intraarticular tibiofemoral; ICC, intraclass correlation coefficient; MRI, magnetic resonance imaging.

**Table 3:** Epidemiological data of the included patients.

Patient demographics	
Number	78
Age (y)	25.6 ± 9.6 (12–53)
Male (*n*)	17 (21.8%)
Female (*n*)	61 (78.2%)

*Note*: Results are presented as number (%) or mean ± standard deviation (SD) (min.–max.).

**Table 4:** Mean values of iaTF rotation, femoral torsion and tibial torsion measured in torsional MRI and iaTF rotation, degree of flexion in the sagittal plane and depicted femoral and tibial length in routine MRI.

MRI analysis	Median [minimum; maximum](mean ± SD)
Torsional MRI	
iaTF rotation (°)	5.4 [−7.4; 36.5] (5.7 ± 6.7)
Femoral torsion (°)	−33.9 [−67.2; 10.7] (−33.0 ± 12.2)
Tibial torsion (°)	42.0 [20.3; 65.7] (42.0 ± 8.5)
Routine MRI	
iaTF rotation (°)	1.2 [−17.6; 21.8] (1.0 ± 8.0)
Flexion (°)	7.0 [−7.9; 23.0] (7.1 ± 6.1)
Femoral lenght (mm)	89.2 [58.1; 386.1] (93.5 ± 34.2)
Tibial lenght (mm)	71.2 [18.2; 115.6] (71.8 ± 13.8)

Abbreviations: iaTF, intraarticular tibiofemoral; MRI, magnetic resonance imaging; SD, standard deviation.

### Direct comparison of iaTF rotation measured in torsional and routine MRI

**Figure 2:**
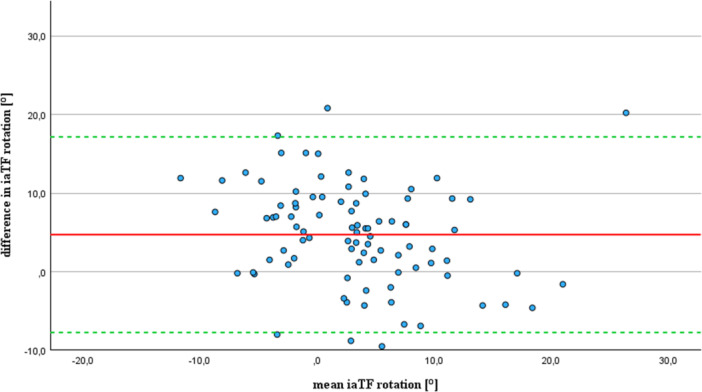
Bland–Altman Plot for consistency of iaTF rotation measured in torsional and routine MRI. Mean iaTF rotation was calculated individual for each case from the values measured in torsional and routine MRI. The difference was also calculated case by case by subtracting the iaTF rotation from the routine MRI from that from the torsional MRI. Cases in which iaTF rotation was greater in routine MRI than in torsional MRI (*n* = 22) are associated with negative values. The red line indicates the mean difference in iaTF rotation (including negative values) and is 4.7°. Green dashed lines marking the 1.96‐fold standard deviation (upper limit: 17.1°, lower limit: −7.7°) [4, 25]. iaTF, intraarticular tibiofemoral; MRI, magnetic resonance imaging.

The iaTF rotation deviated by an absolute value of 5.8 [0.1–20.8] (6.4° ± 4.6°) between torsional MRI and routine MRI. The correlation analysis revealed a moderate positive and significant correlation between the results of the two imaging modalities (*r*_s_ = 0.591; *p* < 0.001) while the intraclass correlation coefficient of 0.688 [0.270–0.842] showed a moderate agreement between the values of torsional and routine MRI. In the Bland–Altman plot (Figure 2), the average iaTF rotation from torsional and routine MRI was calculated for each patient and plotted against the difference between these two measurements. The limits were calculated on −7.7° and 17.1°.

### The influence of flexion measured in the routine MRI on the iaTF rotation

**Figure 3:**
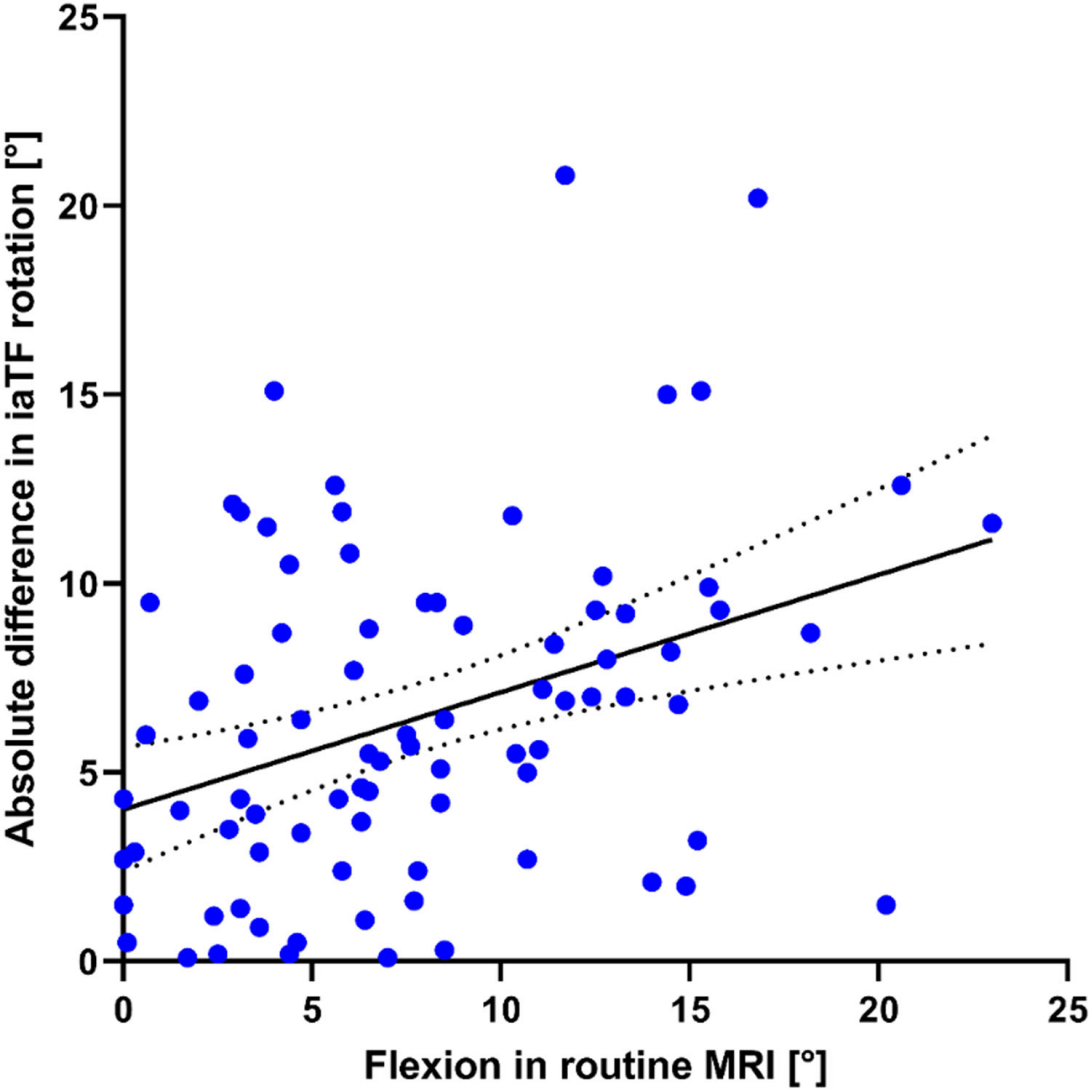
Correlation between flexion measured in routine MRI (x‐axis) and the absolute difference in iaTF rotation between torsional and routine MRI (y‐axis). The blue dots represent individual cases. The black solid line represents the best fit line according to spearman correlation analysis. iaTF, intraarticular tibiofemoral; MRI, magnetic resonance imaging.

Six cases of routine MRI were excluded from the analysis in the following section (Figure 3) due to implausible negative flexion values as explained in Section Methods, METHODS.

The extent of the difference in iaTF rotation measured in torsional and routine MRI is weakly positively correlated with the flexion measured in routine MRI (*r*_s_ = 0.349, *p* = 0.001). Figure 3 illustrates this relationship for the individual case pairs. No correlation was found between the difference in iaTF rotation and the imaged length of femur (*r*_s_ = −0.019; *p* = 0.867) and tibia (*r*_s_ = −0.065; *p* = 0.554). In 22 cases, the measured iaTF rotation deviated by 3° or less from routine MRI compared to torsional MRI. For these cases alone the intraclass correlation coefficient for the measured iaTF rotation between torsional and routine MRI was considered excellent being 0.986 [0.962–0.995] as well the Spearman correlation was very strong (*r*_s_ = 0.958; *p* < 0.001). Comparing this 'measured difference ≤ 3°'‐ group with the remaining 66 cases, there was a significant difference in the degree of flexion measured in routine MRI with the 'measured difference ≤ 3°'‐ group showing significantly lower flexion values (*p* = 0.022). No difference was found regarding the femoral and tibial lengths.

**Figure 4:**
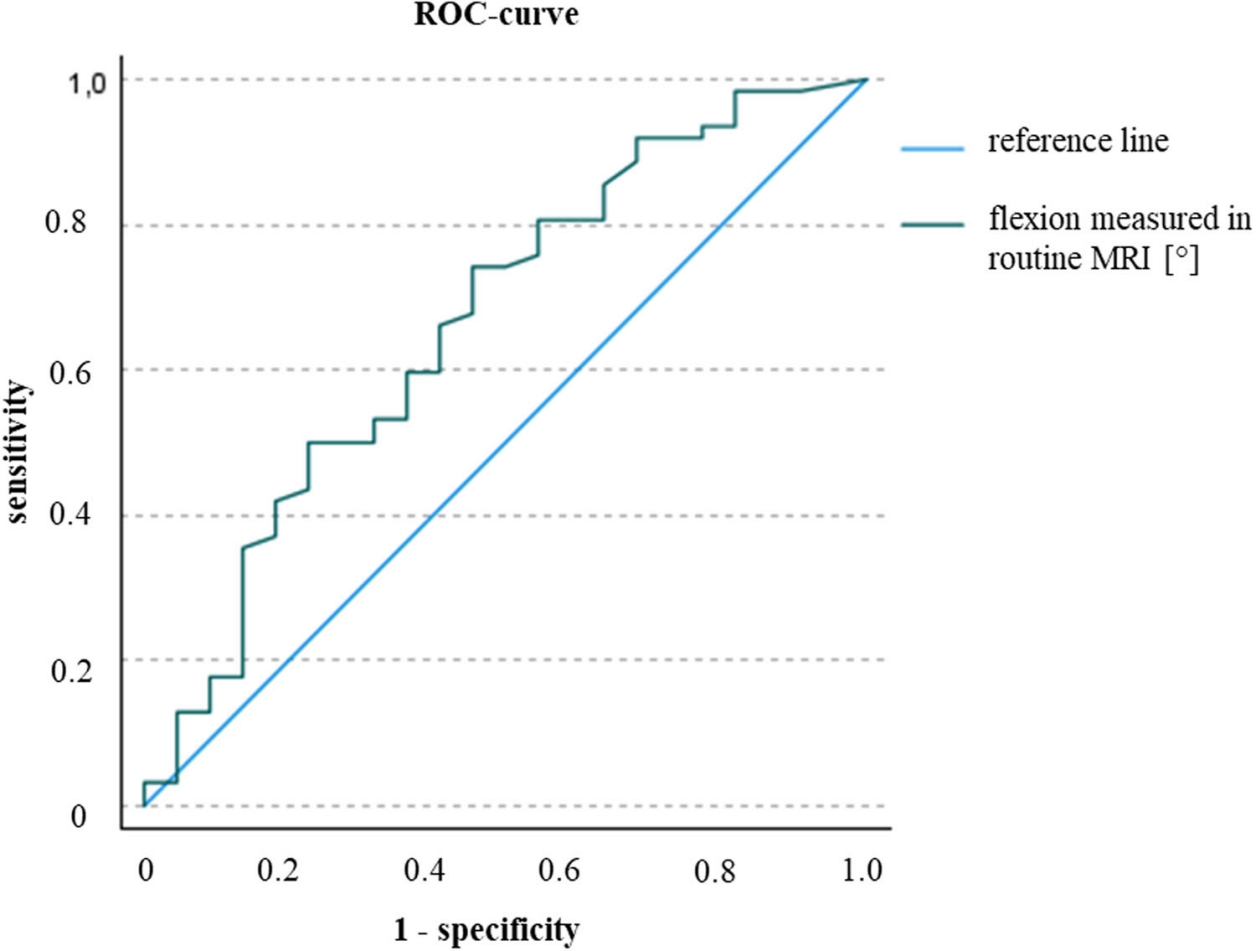
ROC curve to determine the assignability of the iaTF rotation measurements to the 'measured difference ≤ 3°'‐group based on the flexion measured in the routine MRI. iaTF, intraarticular tibiofemoral; MRI, magnetic resonance imaging.

Assuming that the measured flexion in routine MRI has an influence on the concurrence between torsional and routine MRI, the cases were divided into the 'measured difference ≤ 3°' group and the remaining cases (as in Table 5). On this basis, a ROC curve was plotted (Figure 4). The overall model quality of the ROC analysis was 0.53 with the threshold set on 4.65° flexion. For cases with a flexion being 4.65° or less a sensitivity of 74.2% and a specificity of 54.5% resulted for the prediction of being in the 'measured difference ≤ 3°' group.

**Table 5:** Comparison of cases with a good agreement of iaTF rotation in torsional and routine MRI with cases having an insufficient agreement.

iaTF rotation: Agreement between torMRI and rouMRI	Cases with good agreement (difference ≤ 3°)	Cases with insufficient agreement (difference > 3°)	*p*‐Value
*n*	22	62	
Mean absolute difference (°)	1.5 [0.1; 2.9] (1.4 ± 1.0)	7.6 [3.2; 20.8] (8.3 ± 3.8)	<0.001
iaTF rotation torMRI (°)	5.5 [−6.9; 20.2] (5.2 ± 7.2)	6.0 [−7.4 – 36.5] (6.1 ± 6.8)	0.799
iaTF rotation rouMRI (°)	4.8 [−6.7; 21.8] (4.5 ± 7.4)	−0.9 [−17.6; 20.7] (−0.3 ± 7.9)	0.010^#^
Flexion (°)	4.5 [0.0; 20.2] (5.9 ± 5.3)	7.8 (0.0; 23.0) (8.6 ± 5.2)	0.022
Femoral length (mm)	86.9 [58.1; 109.8] (86.9 ± 12.4)	89.9 [66.4; 386.1] (96.6 ± 40.2)	0.236
Tibial length (mm)	73.3 [54.8; 94.8] (73.4 ± 11.8)	72.5 [18.2; 115.6] (72.4 ± 14.5)	0.907

*Note*: A good agreement was considered for cases with a difference ≤ 3° between imaging modalities. Mean absolute difference is calculated as iaTF rotation in torsional MRI ‐ iaTF rotation in routine MRI and the absolute value was taken. The statistical comparisons were performed using the Mann–Whitney *U* test. Exceptions where data was normal distributed are marked with #. A t‐test was conducted here.

Abbreviations: iaTF, intraarticular tibiofemoral; MRI, magnetic resonance imaging; rouMRI, routine MRI; torMRI, torsional MRI.

## DISCUSSION

The most important finding of the present study is that iaTF rotation measurements differed by an absolute value of 5.8° [0.1°–20.8] between torsional MRI and routine MRI.

Thus, the initial hypothesis was confirmed. The statistical analysis of the data determined that it is not reliable to measure iaTF rotation in most routine MRIs. This statement was supported by the following results. The Spearman correlation coefficient between the measured values in torsional and routine MRI demonstrated just in a moderate linear correlation. The intraclass correlation coefficient between the iaTF rotation measured in torsional and routine MRI showed only moderate reliability with ICC 95%‐confidence interval ranging from poor to good reliability [20]. The Bland–Altman plot also showed a poor agreement between torsional and routine MRI regarding iaTF rotation. The calculated limits were 24.8° apart. Essentially, Spearman's correlation, ICC, and Bland‐Altman plot provide a clear indication for the insufficient agreement between routine MRIs and torsional MRIs. Thus, using a routine MRI instead of a torsional MRI does not seem acceptable for clinical use.

In a recent study on iaTF rotation measurement in torsional CT compared to routine MRI, Jud et al. determined a mean difference of 4.6° ± 4.6° between imaging modalities [17]. They were able to demonstrate a positive correlation between the degree of flexion and the difference in iaTF rotation [17]. The present study confirmed those findings. However, further statistical evaluation revealed that a low degree of subsequently measured flexion in routine MRI is not sufficient for a reliable determination of the iaTF rotation. Therefore, the secondary hypothesis had to be rejected, based on the ROC analysis, which revealed a poor overall model quality (0.53) and low sensitivity (74.2%) and specificity (54.5%).

A change in the iaTF rotation with increasing degree of flexion is likely to be explained by the screw‐home mechanism [28]. A similar effect was shown for tibial tubercle to trochlea groove (TT‐TG) distance, which decreases significantly with the degree of flexion [1, 2, 6, 7, 9, 24] and shows a moderate positive correlation with iaTF rotation [13]. As iaTF rotation, TT‐TG shows lower values when measuring is performed on routine MRI compared to CT or torsional MRI [12, 32]. A similarity between the behaviour of TT‐TG and iaTF rotation seems likely, as Pascual‐Leone et al. demonstrated in a recent study that both, TT‐TG and iaTF rotation, show significant differences between MRI examinations at different time points and that the differences correlate significantly with each other [27]. Calmathias et al. were able to show in a cadaver study that a change in intra‐articular rotation has a significant effect on TT‐TG [6].

Interestingly, Ackermann et al. were able to show that a higher knee rotation angle is negatively correlated with the difference between TT‐TG measured in MRI and CT [1]. Given the relationship between TT‐TG and iaTF rotation described above, this is relevant for interpreting the results of the present study. Due to the inclusion criteria, patients in the present study showed higher iaTF rotation compared to healthy individuals [10, 16]. Based on the findings of Ackerman et al. [1]and assuming a positive correlation between TT‐TG and iaTF rotation [13], the values from torsional MRI and routine MRI should show greater agreement with higher iaTF rotation than with normal iaTF rotation values. Vice versa, this would mean that in healthy individuals, the iaTF rotation between torsional MRI and routine MRI would differ even more.

The median degree of flexion measured in routine MRI in the present study (7.0° [−7.9°; 23.0°]) is in line with the values from Griffith et al. among their healthy control group with extended knee joints (7.7° ± 3.4°) and higher than the 2.4° ± 3.1° flexion measured by Jud et al. in fully extended knees in torsional CT scans [17]. Even if a reliable measurement of knee flexion in the sagittal plane in MRI is possible according to the literature [31], the results presented here raise the question of whether the sagittal flexion measurement is able to represent the actual flexion values of the knee joint or whether the values are much more likely to be caused by the individual antecurvation of the distal femur or the shape of the proximal tibia. Therefore, such flexion values measured retrospectively on routine MRI scans should be interpreted with caution.

## LIMITATIONS

This study is not without limitations. First, since there was no uniform protocol for routine MRI examinations, the degree of joint flexion could only be measured subsequently within the MRI images – it is conceivable that this measurement focused more on bone morphology than on the actual degree of flexion in the knee joint, which is why even negative flexion values were measured. It would have been interesting to compare the degree of flexion measured clinically with the measurements based on imaging. Second, the present study was conducted on patients with a patellofemoral pathology and a torsional deformity who had an indication for surgery. This could have an unknown influence on the transferability of the results to individuals without a torsional deformity. Furthermore, routine MRIs are usually performed in a more acute setting then torsional MRI analysis, so a joint effusion may influence iaTF rotation. The Spearman correlation analysis performed is not an ideal statistical test for comparing two measurement methods, as a strong positive linear correlation can also occur if one measured value is constantly higher than the other [4, 5, 25]. Nevertheless, this method is mentioned here because the calculated correlation coefficient is just in the lower range of a strong linear correlation and therefore already indicates that there does not appear to be sufficient agreement between the measurements in torsional and routine MRI.

## CONCLUSION

A measurement of the iaTF rotation using routine MRIs with no uniform protocol cannot be recommended, as the results deviate considerably from those in torsional MRIs. Even by including knee joint flexion measured subsequently in routine MRI, it was not possible to achieve a good agreement between the two imaging modalities.

## AUTHOR CONTRIBUTIONS

**Sina Gräber**: Conceptualisation; measurements; statistical analysis; writing – original draft preparation. **Annett Klinder**: Statistical analysis; writing – review and editing. **Johanna Lippuner**: Additional measurements as part of the revision process; writing – review and editing. **Felix Hüttner**: measurements; writing – review and editing. **Andrzej Jasina**: Writing – review and editing. **Parisa Pourostad**: Writing – review and editing. **Elizabeth Arendt**: Writing – review and editing. **Thomas Tischer**: Writing – review and editing. **Jörg Harrer**: Data provision, conceptualisation; writing – review and editing. **Felix Ferner**: Data provision; conceptualisation; writing – review and editing. **Christoph Lutter**: Conceptualisation, writing – review and editing; supervision.

## CONFLICT OF INTEREST STATEMENT

Thomas Tischer is a member of the editorial board of the Journal of Experimental Orthopaedics (JEO). All other authors declare no conflict of interest.

## FUNDING INFORMATION

The authors have no funding to report.

## ETHICS STATEMENT

Ethical approval (A2020‐0273) was waived by the local Ethics Committee from Rostock University Medical Center in view of the retrospective nature of the study and all the procedures being performed were part of the routine care.

## Data Availability

The data that support the findings of this study are available on request from the corresponding author. The data are not publicly available due to privacy and ethical restrictions.
